# Probable Non–Vector-borne Transmission of Zika Virus, Colorado, USA

**DOI:** 10.3201/eid1705.101939

**Published:** 2011-05

**Authors:** Brian D. Foy, Kevin C. Kobylinski, Joy L. Chilson Foy, Bradley J. Blitvich, Amelia Travassos da Rosa, Andrew D. Haddow, Robert S. Lanciotti, Robert B. Tesh

**Affiliations:** Author affiliations: Colorado State University, Fort Collins, Colorado, USA (B.D. Foy, K.C. Kobylinski);; Poudre Valley Hospital, Fort Collins (J.L.C. Foy);; Iowa State University, Ames, Iowa, USA (B.J. Blitvich);; University of Texas Medical Branch, Galveston, Texas, USA (A. Travassos da Rosa, A.D. Haddow, R.B. Tesh);; Centers for Disease Control and Prevention, Fort Collins (R.S. Lanciotti)

**Keywords:** Zika virus, arbovirus, flavivirus, viruses, mosquitoes, non–vector-borne transmission, sexually transmitted infection, Colorado, expedited, dispatch

## Abstract

Clinical and serologic evidence indicate that 2 American scientists contracted Zika virus infections while working in Senegal in 2008. One of the scientists transmitted this arbovirus to his wife after his return home. Direct contact is implicated as the transmission route, most likely as a sexually transmitted infection.

Zika virus (ZIKV), a mosquito-transmitted flavivirus, has been isolated from sentinel monkeys, mosquitoes, and sick persons in Africa and Southeast Asia (*1**,**2*). Serologic surveys indicate that ZIKV infections can be relatively common among persons in southeastern Senegal and other areas of Africa, but that ZIKV-associated disease may be underreported or misdiagnosed. In 2007, a large outbreak of ZIKV infection occurred on Yap Island in the southwestern Pacific that infected ≈70% of the island’s inhabitants (*3*), which highlighted this virus as an emerging pathogen. The purpose of this study was to investigate and report 3 unusual cases of arboviral disease that occurred in Colorado in 2008.

## The Study

Two American scientists (patients 1 and 2) lived and worked in the village of Bandafassi in southeastern Senegal in August 2008 while performing a mosquito-sampling project in surrounding villages. Patients 1 and 2 were men (36 and 27 years of age, respectively), and both had received the yellow fever 17D vaccine before their travel to Senegal. During their project, both patients reported being bitten often by wild *Aedes* spp. mosquitoes in the evenings while they worked. Patients 1 and 2 left Bandafassi on August 21, stayed in Dakar for 2 days, and then returned to their homes in northern Colorado on August 24. Both patients became ill 6–9 days after their return.

Symptoms in patient 1 began on August 30 and consisted of swollen ankles, a maculopapular rash on his torso, and extreme fatigue and headache, but no fever was recorded. On August 31, he experienced the same symptoms and light-headedness and chills, wrist and ankle arthalgia, and symptoms of prostatitis (perineal pain and mild dysuria). However, he remained afebrile. Fatigue and rash decreased on September 1; only residual wrist arthralgia, headache, and prostatic symptoms persisted. On September 2, two aphthous ulcers appeared on his lip. On September 3, he and his wife observed signs of hematospermia (red–brown fluid in his ejaculate) that lasted until September 7. Patient 2 experienced his symptoms during August 29–September 1, which included a maculopapular rash on his torso, extreme fatigue, headache, and swelling and arthralgia in his wrists, knees, and ankles. However, symptoms of prostatitis or hematospermia did not devlop. Acute-phase blood samples were obtained from both patients on September 2.

In patient 3 (a nurse and the wife of patient 1) similar clinical symptoms developed on September 3, including malaise, chills, extreme headache, photophobia, and muscle pain that continued through September 6. She did not have detectable fever. On September 7, a maculopapular rash developed on her torso (Figure) that expanded to her neck and thighs on the following day, and an aphthous ulcer developed on her inside lip. On September 8, arthralgia in her wrists and thumbs and conjunctivitis developed. Her acute symptoms waned over the next several days. Patient 3 had an acute-phase blood sample drawn on September 8. On September 11, she visited her primary care physician, who performed a complete blood count test and studies of hepatic function; all results were within reference ranges. Patient 2 reported wrist arthralgia for 1 month after his acute illness, and patients 1 and 3 have had recurring wrist or thumb joint arthralgia since their acute illness. Convalescent-phase blood samples were drawn on September 22 from patients 1 and 2 and on September 26 from patient 3.

**Figure:**
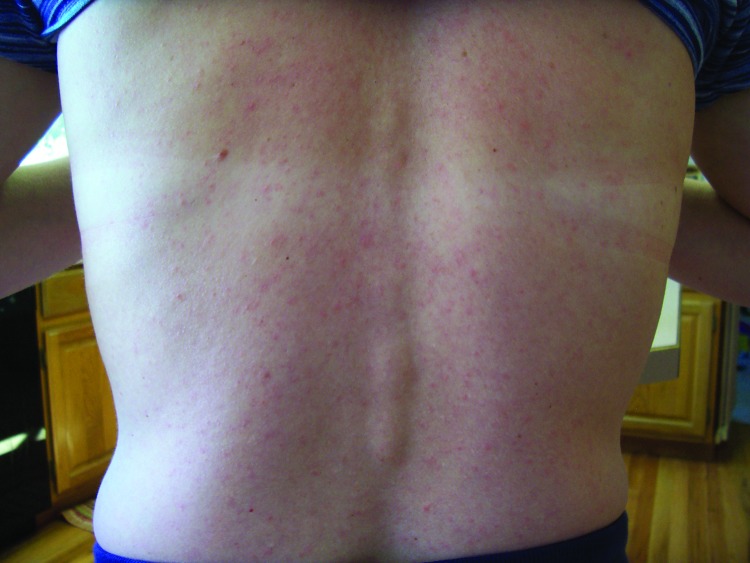
Maculopapular rash on patient 3 infected with Zika virus, Colorado, USA.

Acute-phase and convalescent-phase paired serum specimens from the 3 patients were tested independently by several different laboratories. Results of virus isolation were negative for all samples when tested in Vero and *Aedes albopictus* mosquito (C6/36) cell cultures and by intracerebral inoculation of acute-phase serum of patient 3 into suckling mice. Likewise, reverse transcription–PCRs with 16 different sets of arbovirus-specific primers did not detect arboviral RNA in any of the samples. Serologic analyses (Table A1) of samples from patients 1 and 2 showed matching results. Hemagglutination inhibition antibody titers and virus neutralizing titers were highly elevated above background levels for ZIKV and yellow fever virus (YFV) compared with other viruses tested. These titers most often increased in the time between obtaining acute-phase and convalescent-phase serum samples. Complement fixation tests against ZIKV and YFV antigens confirmed these interpretations. Hemagglutination inhibition, complement fixation, and virus neutralizing titers against ZIKV alone developed only in the convalescent-phase sample of patient 3.

## Conclusions

Evidence suggests that patients 1 and 2 were infected with ZIKV, probably in southeastern Senegal, by bites from infected mosquitoes. The village of Bandafassi is located in a disease-endemic area where ZIKV has been isolated from humans, nonhuman primates, and mosquitoes (*4**,**5*). Serologic results suggest an anamnestic response to ZIKV infection, likely stemming from their vaccination with YFV. The time between infection and the onset of clinical manifestations can be inferred to be >9 days, given the patients’ travel history. Their clinical symptoms are consistent with reported symptoms of ZIKV-associated disease (*3**,**6**–**9*). Exceptions are aphthous ulcers in patient 1 (also reported by patient 3), prostatitis, and hematospermia. Whether these exceptions are typical but unreported symptoms or clinical anomalies is not clear.

Results also support ZIKV transmission from patient 1 to patient 3. Patient 3 had never traveled to Africa or Asia and had not left the United States since 2007. ZIKV has never been reported in the Western Hemisphere. Circumstantial evidence suggests direct person-to-person, possibly sexual, transmission of the virus. Temperatures and mosquito fauna on the northern Front Range in Colorado when transmission occurred do not match known mosquito transmission dynamics of ZIKV by tropical *Aedes* species. Patient 3 had ZIKV disease 9 days after the return of her husband from Senegal. However, the extrinsic incubation period of ZIKV in *Ae. aegypti* mosquitoes was >15 days at 22°C–26°C (*10*). Area temperatures during the week of return of patient 1 fluctuated between 10°C and 31°C, only *Ae. vexans* mosquitoes of the subgenus *Aedimorphus* are commonly captured on the northern Colorado Front Range, and known tropical ZIKV vectors are mostly from the subgenus *Stegomyia* (*4*). Mosquito sampling around the home of patients 1 and 3 at the time yielded only 7 *Ae. vexans* mosquitoes and 11 other mosquitoes of the *Culex* and *Culiseta* genera.

Furthermore, patients 1 and 3 reported having vaginal sexual intercourse in the days after patient 1 returned home but before the onset of his clinical illness. It is reasonable to suspect that infected semen may have passed from patient 1 to patient 3 during coitus. Another possibility is that direct contact and exchange of other bodily fluids, such as saliva, could have resulted in ZIKV transmission, but illness did not develop in the 4 children of patients 1 and 3 during this time.

To the best of our knowledge, human sexual transmission of an arbovirus has not been documented. However, Japanese encephalitis virus was discharged into the semen of experimentally infected boars and could infect female pigs by artificial insemination (*11*). Furthermore, West Nile virus RNA and St. Louis encephalitis virus antigen have been detected in urine of humans (*12**,**13*), and viruria has occurred in hamsters infected with West Nile virus (*14*) and Modoc virus (*15*). If sexual transmission could be verified in subsequent studies, this would have major implications toward the epidemiology of ZIKV and possibly other arthropod-borne flaviviruses.

## Appendix

**Table A1:** Serologic results for 3 patients in whom symptoms of arboviral illness developed, northern Colorado, USA, 2008*

Serum sample	Viruses/viral antigens tested														
	GETV	CHIKV	SINV	ZIKV	DENV1	DENV2	DENV3	DENV4	YFV	WNV	UGSV	SLEV	TAHV	JCV	LACV
Hemagglutination inhibition test															
Pt1a	0	0	NT	5,120	2,560	640	2,560	2,560	5,120	2,560	1,280	NT	0	NT	NT
Pt1c	0	0	NT	5,120	1,280	320	1,280	1,280	2,560	1,280	1,280	NT	0	NT	NT
Pt2a	0	0	NT	1,280	160	80	320	320	1,280	160	160	NT	0	NT	NT
Pt2c	0	0	NT	2,560	160	80	320	320	1,280	160	160	NT	0	NT	NT
Pt3a	0	0	NT	0	0	0	0	0	0	0	0	NT	0	NT	NT
Pt3c	0	0	NT	320	0	0	0	0	0	0	0	NT	0	NT	NT
Plaque-reduction neutralization test															
Pt1a	NT	<10	<10	**10,240**	80	**1,280**	320	160	**>20,480**	<10	NT	80	<10	NT	NT
Pt1c	NT	NT	NT	**>40,960**	160	230	320	320	**>40,960**	160	NT	NT	NT	NT	NT
Pt2a	NT	NT	NT	**10,240**	<20	80	80	40	**5,120**	NT	NT	NT	NT	NT	NT
Pt2c	NT	NT	NT	**40,960**	80	160	160	160	**20,480**	80	NT	NT	NT	NT	NT
Pt3a	NT	NT	NT	10	<10	<10	<10	<10	<10	<10	NT	<10	NT	<10	<10
Pt3c	NT	NT	NT	**1,280**	<10	<10	<10	<10	<10	<10	NT	NT	NT	NT	NT
Complement fixation test															
Pt1a	NT	NT	NT	64/8	NT	NT	NT	NT	64/8	NT	NT	NT	NT	NT	NT
Pt1c	NT	NT	NT	128/32	NT	NT	NT	NT	64/8	NT	NT	NT	NT	NT	NT
Pt2a	NT	NT	NT	16/8	NT	NT	NT	NT	16/8	NT	NT	NT	NT	NT	NT
Pt2c	NT	NT	NT	32/32	NT	NT	NT	NT	16/8	NT	NT	NT	NT	NT	NT
Pt3a	NT	NT	NT	0	NT	NT	NT	NT	0	NT	NT	NT	NT	NT	NT
Pt3c	NT	NT	NT	16/8	NT	NT	NT	NT	0	NT	NT	NT	NT	NT	NT

**Togaviridae*: GETV, Getah virus; CHIKV, chikungunya virus; SINV, Sindbis virus; *Flaviviridae*: ZIK, Zika virus; DENV, dengue virus; YFV, yellow fever virus; WNV, West Nile virus; UGSV, Uganda S virus; SLEV, St. Louis encephalitis virus; *Bunyaviridae*: TAHV, Tahyna virus; JCV, Jamestown Canyon virus; LAC, La Crosse virus; pt1, 2, and 3, patients 1, 2, and 3, respectively; a and c, acute-phase and convalescent-phase serum samples, respectively; NT, not tested. Hemagglutination test, numbers are reciprocal of serum dilution inhibiting 4 units of antigen; 0 = negative reaction at a 1:20 dilution. Plaque-reduction neutralization test, numbers refer to neutralizing antibody titers with a 90% cutoff value: titers > positive control serum samples are indicated in **boldface**. Complement fixation tests, numbers refer to reciprocal of serum titer/reciprocal of antigen titer; 0, negative reaction at <8/<8.
